# A novel starch-binding laccase from the wheat pathogen *Zymoseptoria tritici* highlights the functional diversity of ascomycete laccases

**DOI:** 10.1186/s12896-019-0552-4

**Published:** 2019-08-19

**Authors:** Majid Haddad Momeni, Paolo Bollella, Roberto Ortiz, Esben Thormann, Lo Gorton, Maher Abou Hachem

**Affiliations:** 1 Department of Biotechnology and Biomedicine, Technical University of Denmark, Søltofts Plads, 2800 Kgs, Lyngby, Denmark; 2 Department of Biochemistry and Structural Biology, Lund University, P.O. Box 124, 221 00 Lund, Sweden; 3 Department of Chemistry and Drug Technologies, Sapienza University of Rome, Piazzale Aldo Moro 5, 00185 Rome, Italy; 4 Department of Chemistry, Technical University of Denmark, Kemitorvet 207, 2800 Kgs, Lyngby, Denmark

**Keywords:** Carbohydrate binding module family 20 (CBM20), Cyclic voltammograms, Laccase, Oxidoreductase, Plant, Pathogen, Starch, *Zymoseptoria tritici*

## Abstract

**Background:**

Laccases are multicopper oxidases, which are assigned into auxiliary activity family 1 (AA1) in the CAZy database. These enzymes, catalyzing the oxidation of phenolic and nonphenolic substrates coupled to reduction of O_2_ to H_2_O, are increasingly attractive as eco-friendly oxidation biocatalysts. Basidiomycota laccases are well characterized due to their potential in de-lignification of lignocellulose. By contrast, insight into the biochemical diversity of Ascomycota counterparts from saprophytes and plant pathogens is scarce.

**Results:**

Here, we report the properties of the laccase from the major wheat pathogen *Zymoseptoria tritici* (*Ztr*Lac1A), distinguished from common plant fungal pathogens by an apoplastic infection strategy. We demonstrate that *Ztr*Lac1A is appended to a functional starch-binding module and displays an activity signature disfavoring relatively apolar phenolic redox mediators as compared to the related biochemically characterized laccases. By contrast, the redox potential of *Ztr*Lac1A (370 mV vs. SHE) is similar to ascomycetes counterparts. The atypical specificity is consistent with distinctive sequence substitutions and insertions in loops flanking the T1 site and the enzyme C-terminus compared to characterized laccases.

**Conclusions:**

*Ztr*Lac1A is the first reported modular laccase appended to a functional starch-specific carbohydrate binding module of family 20 (CBM20). The distinct specificity profile of *Ztr*Lac1A correlates to structural differences in the active site region compared to previously described ascomycetes homologues. These differences are also highlighted by the clustering of the sequence of *Ztr*Lac1A in a distinct clade populated predominantly by plant pathogens in the phylogenetic tree of AA1 laccases. The possible role of these laccases in vivo merits further investigations. These findings expand our toolbox of laccases for green oxidation and highlight the binding functionality of CBM-appended laccases as versatile immobilization tags.

**Electronic supplementary material:**

The online version of this article (10.1186/s12896-019-0552-4) contains supplementary material, which is available to authorized users.

## Background

Laccases (EC 1.10.3.2) are multicopper oxidases (MCOs), which catalyze monoelectric oxidation of a variety of substrates using molecular oxygen that is reduced to water [1–3]. A mononuclear copper binding site designated as a type-1 Cu (blue Cu) and a trinuclear copper site, involving one type-2 and two type-3 Cu (coupled binuclear Cu) binding sites are typically present in laccases. Substrates are oxidized in vicinity of the high redox potential mononuclear site (Cu1 or T1), which is responsible for the blue color and distinctive absorbance of these enzymes at about 600 nm [1, 4, 5]. Thus, the type-1 Cu possesses a higher redox potential as compared to its counterparts in the type 2 and type 3 sites [6]. The electrons from the oxidation of substrates at the T1 site are transferred through the protein via the Cys-His pathway to the trinuclear cupper cluster at the T2/T3 sites, where the electrons are transferred to oxygen (O_2_) [5].

**Fig. 1:**
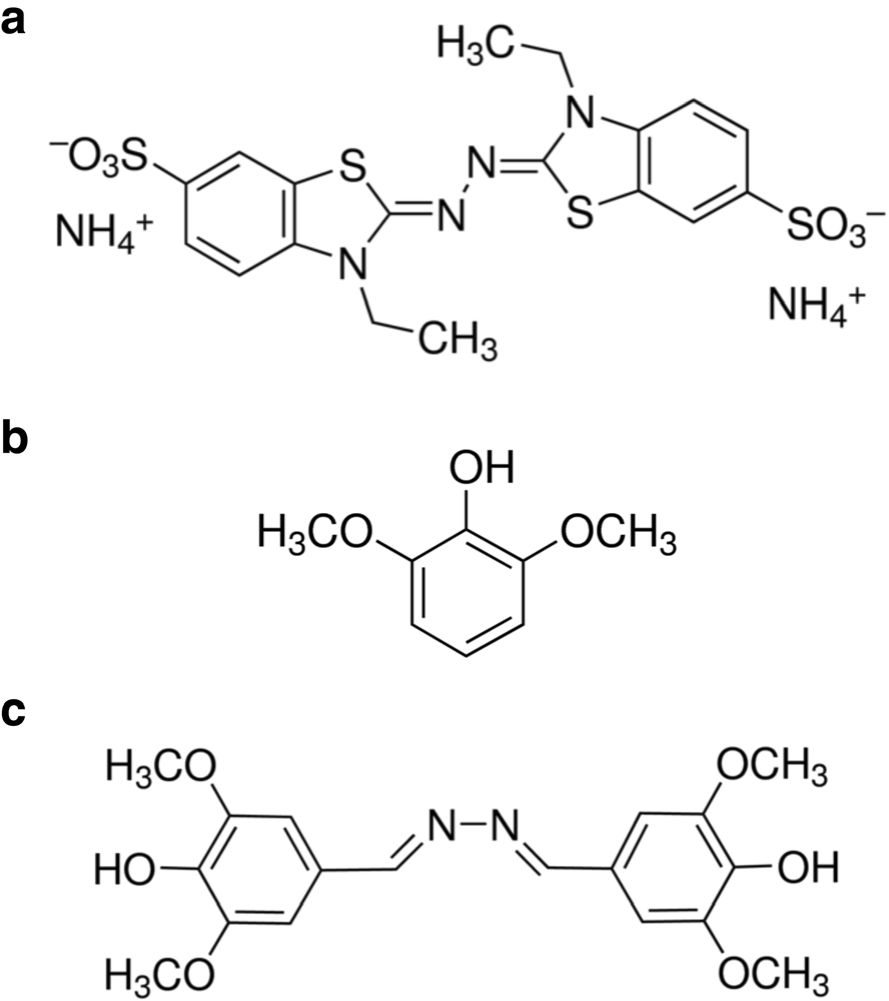
Chemical structures of three synthetic redox mediators**, a** 2,2-azino-bis (3-ethylbenzothiazoline-6-sulfonic acid) diammonium salt, ABTS (**b**) 2,6 dimethoxyphenol, DMP (**c**) syringaldazine

Laccases are considered as eco-friendly catalysts due to their ability to oxidize a broad range of organic and synthetic substrates using only oxygen as the electron acceptor with no toxic byproducts. Some potential substrates are large in size precluding their accommodation into the active sites of laccases. Small redox mediators, e.g. 2,2-azino-bis(3-ethylbenzothiazoline-6-sulfonic acid) diammonium salt (ABTS), 2,6 dimethoxyphenol, DMP and syringaldazine (Fig. 1), generally act as electron shuttles that mediate the oxidation of substrates too large for the direct oxidation.

Although, laccases are produced by diverse taxonomic groups including bacteria, insects, lichens, plants and fungi, the latter category mainly dominates in biotechnological applications. The wide utilization of fungal laccases in biotechnological applications is likely attributed to the stability, industrial efficient heterologous expression systems and high redox potential of Basidiomycota enzymes [3, 7]. Particularly, the abundance of laccases in almost all wood-rotting fungi has attracted interest in the context of de-lignification of lignocellulose, but laccases are also implicated in lignification, oxidative stress management in plants and are frequently active on an array of phenolic compounds (e.g. phenols, polyphenols, benzenothiols and anilines) [8].

Fungal laccases are produced by several groups ranging from yeasts, white and brown rot fungi (both from the Basidiomycota phylum), or Ascomycota as well as mycorrhizal species. Laccases are classified into auxiliary activity family 1 (AA1) in the Carbohydrate-Active Enzyme (CAZy) database [9]. Laccases from white rot basidiomycetes are distinguished by their high redox potentials (*E*^*0*^ ≈ 0.8 V for the Cu1 site) [10], as compared to ascomycete and/or bacterial laccases, which display redox potential *E*^*0*^ = 0.4–0.7 V [11]. Notably, the genomes of phytopathogenic ascomycete fungi like e.g. *Botrytis cinera* [12], *Magnaporthe grisea* [13] and *Fusarium oxysporum* [14] each encode several putative laccases of AA1 subfamily 3 (AA1_3), which harbors most ascomycete laccases according to the CAZy classification, whereas basidiomycetes counterpart are assigned into AA1 subfamily 1 (AA_1). Functional insight into AA1_3 laccases from phytopathogens, which may contribute to the understanding of the properties and possible roles of these enzymes during pathogenesis, lags behind.

*Zymoseptoria tritici* (syn. *Mycosphaerella graminicola*, *Septoria tritici*) is responsible for the wheat disease *Septoria tritici* blotch (STB) [15, 16]. This plant disease has been recognized as the most devastating for wheat production in Europe [17, 18], which is also highlighted by the ranking of the causative fungus amongst the top ten fungal pathogens [19]. *Z. tritici* is distinguished by an infection within the apoplastic space (outside the plasma membrane but within the plant cell wall) that differentiates it from other globally recognized fungal phytopathogens e.g. *Fusarium* [20] and *Magnaporthe* spp. [21]*.* The genome of *M. graminicola* IPO323, reveals a significant reduction in the number of carbohydrate active enzymes (CAZymes) that target plant cell walls, especially those possessing a carbohydrate binding module (CBM) [22], consistent with this apoplastic infection strategy. Remarkably, starch-binding modules of CBM family 20 (CBM20) are encoded by four genes from *Z. tritici*: one encoding a hypothetical protein, two encoding putative α-glucosidases and a fourth encoding a laccase.

In this study, we produced and characterized the first modular laccase (hereafter referred to as *Ztr*Lac1A) comprising a catalytic module of AA1_3 and a starch-binding module of CBM20. Curiously, this enzyme has a preference to the negatively charged redox mediator 2,2′-azino-bis(3-ethylbenzothiazoline-6-sulphonic acid) (ABTS) as compared to more apolar mediators. This activity profile is the converse of fungal laccases of AA1_3 characterized to date.

To provide a rationale for this different specificity, we carried out bioinformatic analyses, which revealed marked differences between *Ztr*Lac1A and characterized counterparts in the vicinity of the T1 site, highlighting this region as an important specificity determinant. Moreover, variations in sequence were also observed at the C-terminus, which has been shown to be an important activity signature in laccases. The modularity and atypical specificity profile of *Ztr*Lac1A offer an expansion of our enzymatic tool box of green oxidative enzymes and promote our understanding of the basic features that govern the specificity of AA1 laccases.

## Methods

### Materials and media

ABTS, syringaldazine and 2,6 DMP (2,6-dimethoxyphenol) and other chemicals were from Sigma-Aldrich (St. Louis, MO, USA). Restriction enzymes and molecular biology reagents were from New England Biolabs (Ipswich, MA, United States). All chemicals were of analytical grade. The *Myrothecium verrucaria* bilirubine oxidase (*Mv*BOD, 3.61 mg/mL) was from Novozymes (Bagsværd, Denmark). The osmium polymer ([Os (4,4′-dichloro-2,2′-bipyridine)2(poly-vinylimidazole)10Cl]·Cl, *E*°′ = 0.350 V vs. Ag|AgCl_*sat*_) (Additional file 1: Figure S1) was kindly provided by Prof. Dónal Leech and Dr. Peter Ó Conghaile from Biomolecular Electronics Research Laboratory, National University of Ireland (Galway, Ireland) and prepared as previously reported [23].

The standard yeast selection medium YPDS (Yeast Extract Peptone Dextrose with Sorbitol) comprised 1% yeast extract, 2% peptone, 2% dextrose (glucose), 1 M sorbitol, 2% agar (all w/v, Invitrogen, Carlsbad, CA, USA). The standard yeast expression media BMGY/BMMY (Buffered Glycerol-complex Medium/Buffered Methanol-complex Medium, both from Invitrogen) comprised 1% yeast extract, 2% peptone, 100 mM potassium phosphate, pH 6.0, 1.34% YNB, 4 × 10^−5^% biotin (all w/v), 1% glycerol or 0.5% methanol (both v/v).

### Cloning, expression and purification of recombinant enzyme

The genomic DNA encoding the laccase of auxiliary family 1 [9] (http://www.cazy.org/) from *Zymoseptoria tritici* IPO323 (GenBank accession XP003852363) was synthesized by Invitrogen (Carlsbad, CA, USA). The forward GACAT**TTCGAA**ACGATGCGGTTAC and reverse primer GATTG**TCTAGA**TCAGACTCCGGAATC were used to amplify this gene fragment that encodes the mature peptide of the enzyme lacking the native signal peptide (amino acid residues 46–723, hereafter designated as *Ztr*Lac1A) using *Turbo* DNA polymerase and a PCR protocol involving preheating at 95 °C (30 s), followed by 16 cycles of denaturation at 95 °C (30 s), annealing at 55 °C (1 min) and extension at 68 °C (1 min). The PCR amplicon (2197 bp) was cloned within the BstBI and XbaI restriction sites of *p*PICZαA vector (Invitrogen, Carlsbad, CA, USA) using standard molecular biology protocols. The resulting recombinant *p*PICZαA-*Ztr*lac1A plasmid was transformed into *Escherichia coli* DH5α and clones were selected on low salt LB agar plates supplemented with 25 μg mL^− 1^ Zeocin. Sequencing was performed using the 5′-AOX promoter 3′-AOX terminator universal primers and the internal primers 5′-GGGTTTGAATTATGAGGATCCG-3′ and 3′-TTGACTTGCCAGTAGAGGGTG-5′).

The recombinant plasmid was linearized using PmeI and transformed by electroporation into *Pichia pastoris* X-33 cells according to the manufacturer’s protocol. Transformants were selected on YPDS plates supplemented with 100 μg mL^− 1^ Zeocin according to the manufacturer’s recommendation (EasySelect™ Pichia Expression Kit, Invitrogen) after incubation at 30 °C for 3–5 days. Single transformants were re-streaked on new plates and used to inoculate 5 ml buffered complex medium BMGY. The best secreting transformants were selected based on the SDS-PAGE analysis and activity measurements using ABTS as substrate.

An overnight culture (25 mL, BMGY medium, 30 °C) was grown to *OD*_600_ = 5 prior to inoculation into 0.9 L of the same medium and propagated overnight to OD_*600*_ ≈ 7. Thereafter, cells were harvested by centrifugation (7000 ***g***, 30 min, 4 °C), re-suspended in BMMY to OD_*600*_ ≈ 1, and re-incubated at 30 °C. The culture was supplemented with methanol (0.5% v/v) every 24 h prior to harvesting after 72 h of induction. Culture supernatants were recovered by centrifugation (7500 ***g***, 30 min, 4 °C), pooled, supplemented with (NH_4_)_2_SO_4_ to 0.5 M and incubated for 18–20 h, followed by centrifugation (8000 ***g***, 20 min, 4 °C) and filtration (0.5 μm) prior loading onto a 40 ml β-cyclodextrin-Sepharose affinity column and purification as previously described [24]. The elution fractions containing *Ztr*Lac1A were analyzed using SDS-PAGE and activity measurements according to the protocol described below. Fractions displaying high purity and activity were pooled and concentrated using Amicon filters (MWCO, 30 kDa, Millipore). The concentrated protein loaded onto a HiLoad 36/60 Superdex gel filtration column (GE Healthcare, Uppsala, Sweden) and eluted with 20 mM Na acetate, 150 mM NaCl, pH 5.5 at 0.9 ml min^− 1^. The fractions with highest purity and activity were pooled, concentrated and the protein concentration was determined by measuring the absorbance at 280 nm (*A*_280_) using the theoretical extinction coefficient ε_280_ = 79074 M^− 1^.cm^− 1^ Expasy (http://web.expasy.org/protparam). All Purification steps were performed using an ÄKTA purifier chromatograph (GE Healthcare) at 7 °C. *N*-Glycosylation and *O*-glycosylation sites were predicted using NetNGyc 1.0 (http://www.cbs.dtu.dk/services/NetNGlyc/), and NetOGlyc (http://www.cbs.dtu.dk/services/NetNGlyc/), respectively. To examine *N*-glycosylation, a 9 μl of supernatant was initially denatured at 100 °C (10 min) and furthermore was used in EndoH treatment at 37 °C for 90 min according to the manufacturer’s instructions (New England Biolabs) and finally analyzed using SDS-PAGE.

### Isothermal titration calorimetry

The isothermal titration calorimetry (ITC) experiments were performed using an iTC200 instrument (GE healthcare, Northampton, MA, USA). *Ztr*Lac1A (145 μM) dialyzed against 20 mM NaOAc, pH 5.5 was titrated with 2.1 mM β-cyclodextrin (β-CD) dissolved in the same buffer at 25 °C with an initial injection of 0.4 μL followed by 15 injections of 2 μL. A control titration into the dialysis buffer was used to compensate the heat of dilution. A one-binding site model was fit to the ITC data to determine the equilibrium association constant (*K*_a_), the molar binding enthalpy (Δ*H*) and the stoichiometry of binding (*N*_o_) using the ITC analysis plug in ORIGIN software provided with the instrument.

### Biochemical characterization

The kinetic parameters of *Ztr*Lac1A (*K*_m_ and *k*_cat_) were determined by monitoring the increase in absorbance of oxidized ABTS (*A*_405_, ε_405_ = 36.8 mM^− 1^ cm^− 1^) [25] in 96-well microtiter plates using a PowerWave XS plate reader (BioTeK, Winooski, VT, USA) using eight different concentrations (0.032–4 mM) in 40 mM sodium acetate buffer pH 4.2 at 25 °C. The *Ztr*Lac1A activity as function of pH was measured for 10 min at 25 °C in the pH range 2.2–8.0, using ABTS, as substrate in McIlvaine buffer.

The laccase activity was also determined using 2,6-DMP (*A*_469_, ε_469_ = 19.6 mM^− 1^ cm^− 1^) and syringaldazine (*A*_525_, _ε525_ = 65.0 mM^− 1^.cm^− 1^) in the range of 0.4–8 mM and 0.006–0.11 mM, respectively. The reactions were initiated by the addition of the enzyme to a final concentration of 0.26 μM to pre-temperated mixtures containing substrate and buffer to a final volume of 250 μL. The experiments were carried out in triplicates and the kinetic parameters were determined by fitting the Michaelis-Menten equation to the initial rate data at each substrate concentration using ORIGIN 8.0 (OriginLab, Northampton, MA). An additional activity assay was also carried out using the phenolic substrates coniferyl aldehyde, *p*-coumaric, caffeic acids, vanillin, 3,5 dimethoxybenzoic acid and 3,4 dihydroxybenzoic acid in 2 mM concentrations. All the reactions were performed in 50 mM NaAc pH 5.2 in 0.5 ml reaction volume for 30 and 90 min. The structures of these substrates and their analysis are described in Additional file 1: Figure S2a–e.

### Electrochemical measurements and electrode modification

The redox potential of the T1 site for *Ztr*Lac1A was determined using modified graphite electrode (GE) in the presence and absence of a redox mediator. Electrodes were prepared as previously described for bioelectrochemical studies on multicopper oxidase (MCO) [26]. The spectrographic low-density graphite rods (Ø 3.05 mm, Sigma) were polished by fine emery paper (Turfbak Durite, P1200), thoroughly rinsed with Milli-Q water and dried at room temperature. The GE/Os/ *Ztr*Lac1A (0.16 mM) modified electrodes were prepared by transferring Os-polymer (5 μL, 5 mg mL^− 1^) and freshly prepared poly(ethylene glycol) diglycidyl ether solution (PEGDGE, 2 μL, 10 mg/mL water) to the top of the GE and subsequent incubation for 10 min, followed by adding the enzyme solution (12.6 mg/mL). Thereafter, the modified electrodes were dried at room temperature prior to incubation overnight at 4 °C to complete the cross-linking. Furthermore, in the direct electron transfer approach (in absence of a redox mediator), the same immobilization procedure was also applied but in the absence of the Os-polymer and cross-linking agent. To begin with, the enzyme-modified electrodes were press-fitted into a Teflon holder and rinsed thoroughly with acetate buffer to remove any weakly bound material. All electrode modifications were prepared in triplicate.

A three-electrode cell was used with an Ag|AgCl KCl_sat_ (E = 0.199 V vs. SHE, all redox potentials are reported vs. SHE) as reference electrode and a Pt plate as counter electrode. The laccase modified electrodes were used as working electrode. Cyclic voltammetry measurements were performed in an 0.1 M acetate buffer at pH 4.0 also containing 0.1 M NaClO_4_ at room temperature. Prior to the measurements in the absence of O_2_, N_2_ gas was bubbled through the measuring solution at least for 20 min to maintain O_2_-free conditions. Oxygen saturated conditions were achieved by purging pure O_2_ in the buffer solution for at least 20 min. All cyclic voltammetry measurements were conducted using a potentiostat Metrohm Autolab Potentiostats/Galvanostats (Model PGSTAT128N, Metrohm Autolab B.V., Utrecht, The Netherlands) equipped with Nova 2.1 as software.

### Phylogenetic analysis and homology modelling

Most of the ascomycete laccases (about 90%) assigned into AA1_3 in the CAZy database were retrieved. In addition, the catalytic module of *Ztr*Lac1A was also used as a query in a BLASTp search (https://www.ncbi.nlm.nih.gov/BLAST/) against the non-redundant protein sequence database [27] to retrieve additional basidiomycete sequences within an E-value < 5 × 10^− 71^, sequence identity > 31% and a minimum sequence coverage of 88%. In total 221 sequences from ascomycete and basidiomycetes laccase catalytic modules (AA1) were aligned using MAFFT software standard settings (mafft.cbrc.jp/alignment/). The phylogenic tree, calculated using neighbor joining method with bootstrap value of 1000 in MAFFT server and it was visualized by DENDROSCOPE [28].

A homology model for *Ztr*Lac1A was generated using the laccase structure from *Melanocarpus albomyces* (PDB code 1GW0, 44% sequence identity) as a template and the Schrödinger package. The template enzyme represents the closest structurally and biochemically characterized (Table 1) homologue of *Ztr*Lac1A.

## Results

### Heterologous expression of the modular laccase from *Zymoseptoria tritici* (*Ztr*Lac1A)

The non-codon optimized synthetic gene encoding the modular laccase from *Zymoseptoria tritici* (*Ztr*Lac1A) (Additional file 1: Figure S3a), was cloned into *Pichia pastoris*. The recombinant protein was expressed and purified using β-cyclodextrin-Sepharose affinity chromatography, confirming the binding functionality of the CBM20. The purification was polished with an additional gel filtration step and the yield of pure protein was about 1.5 mg L^− 1^ culture and a specific activity of 45.5 U mg^− 1^. *Ztr*Lac1A migrated as smeary bands with an apparent molecular mass of 93 kDa compared to the theoretically calculated value (79 kDa). The larger size is consistent with *O*- and/or *N*-glycosylation, which are typically observed in recombinant proteins produced in *P. pastoris* [30]. Among seven putative *N*-glycosylation sites in the catalytic module, only three exhibit high score (> 0.6), Asn 209, Asn252 and Asn366. The putative *O*-glycosylation sites were predicted in serine/threonine rich linker regions (Additional file 1: Figure S3a) [31]. Endo-H treatment of the recombinant enzyme resulted in a reduction of about 15 kDa in molecular mass estimated from the migration shift in SDS-PAGE analysis (Additional file 1: Figure S3b) suggesting the protein is mainly decorated with *N*-glycans.

### *Ztr*Lac1A is a functional laccase

The activity of *Ztr*Lac1A was examined using the synthetic mediator ABTS at pH 4.2, whereas activity on syringaldazine and 2,6-DMP was assayed at pH 6.0. Notably, *Ztr*Lac1A is mainly active on ABTS, compared to a negligible activity towards 2,6-DMP and no activity towards syringaldazine, which is different from the closest related ascomycete laccases from *Melanocarpus albomyces* laccase (*Mal*Lac1A) (Table 1).

**Table 1:** Kinetic parameters for *Ztr*Lac1A were determined in triplicates, using 40 mM McIlvaine buffer at pH 4.2 compared to the *Mal*Lac1A using the same substrates

	ABTS	2,6-DMP	Syringaldazine
*Mal*Lac1A^*a*^			
*K*_m_ (mM)	0.4	0.011	0.037
*k*_cat_ (s^−1^)	28.1	10.2	40.2
*k*_cat_/*K*_m_ (s^−1^ mM^- 1^)	70.3	927.3	1085.6
*Ztr*Lac1A			
*K*_m_ (mM)	0.25 ± 0.01	1.00 ± 0.05	ND^b^
*k*_cat_ (s^−1^)	14.0 ± 0.3	0.14 ± 0.00	ND^b^
*k*_cat_/*K*_m_ (s^− 1^ mM^− 1^)	53.3 ± 3.3	0.14 ± 0.01	ND^b^

^*a*^The kinetic parameters for *Mal*Lac1A were recalculated from values measured previously by Andberg et al.*,* using ABTS in 25 mM succinate buffer at pH 4.5, and 2,6-DMP and syringaldazine activities were performed in 40 mM MES buffer at pH 6.0 both at 25 °C. The error in all measurements was estimated to ±15% [29]. ^*b*^ND: Not determined due to lack of activity

### Isothermal titration calorimetry (ITC)

**Fig. 2:**
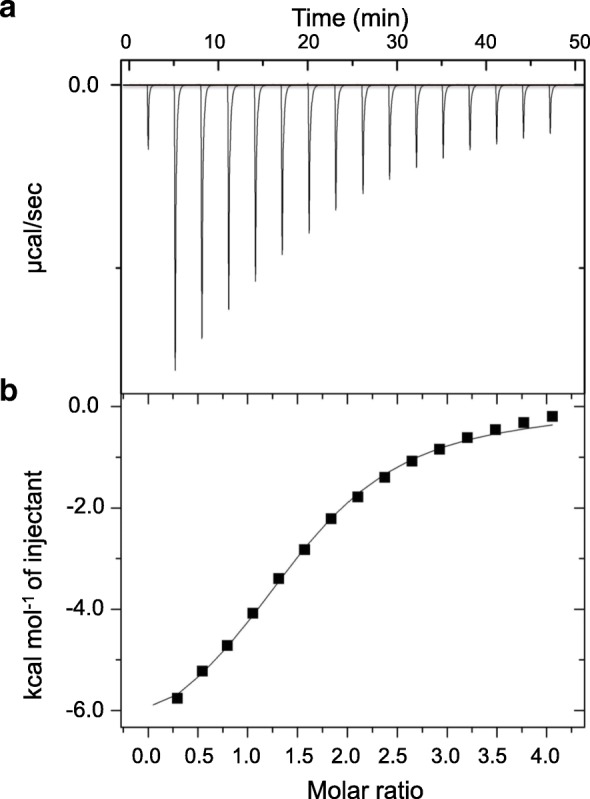
Isothermal titration calorimetry of the binding of *Ztr*Lac1A to β-CD, carried out at 25 °C at pH 5.5. **a** Binding thermogram and **b** The normalized integrated heat response (black squares) plotted against the molar ratio of injected ligand to the protein concentration in the cell. The fit of a one-site binding model (black line) to the binding isotherm

The binding of *Ztr*Lac1A to the starch mimics β-CD, a typical model substrate used to demonstrate the functionality of starch-binding proteins [32], was quantified using ITC (Fig. 2). The data revealed that *Ztr*Lac1A binds to β-CD with an association constant of *K*_a_ = (3.07 ± 0.24) × 10^4^ M^− 1^ (*K*_d_ = 32.6 ± 2.4 μM, Δ*G* = − 6.17 kcal mol^− 1^). The binding was largely driven by a favorable enthalpy (Δ*H* = − 7.15 ± 0.19 kcal mol^− 1^), which is compensated by an unfavorable change in entropy (−*T*Δ*S* = 1.03 kcal mol^− 1^). The fitted stoichiometry of *N*_o_ = 1.5 ± 0.1 is lower, but consistent with the presence of two β-CD binding sites in canonical CBM20 modules [32], which is likely to be due to residual partial occupancy from the β-CD elution during purification.

### Electrochemical characterization

**Fig. 3:**
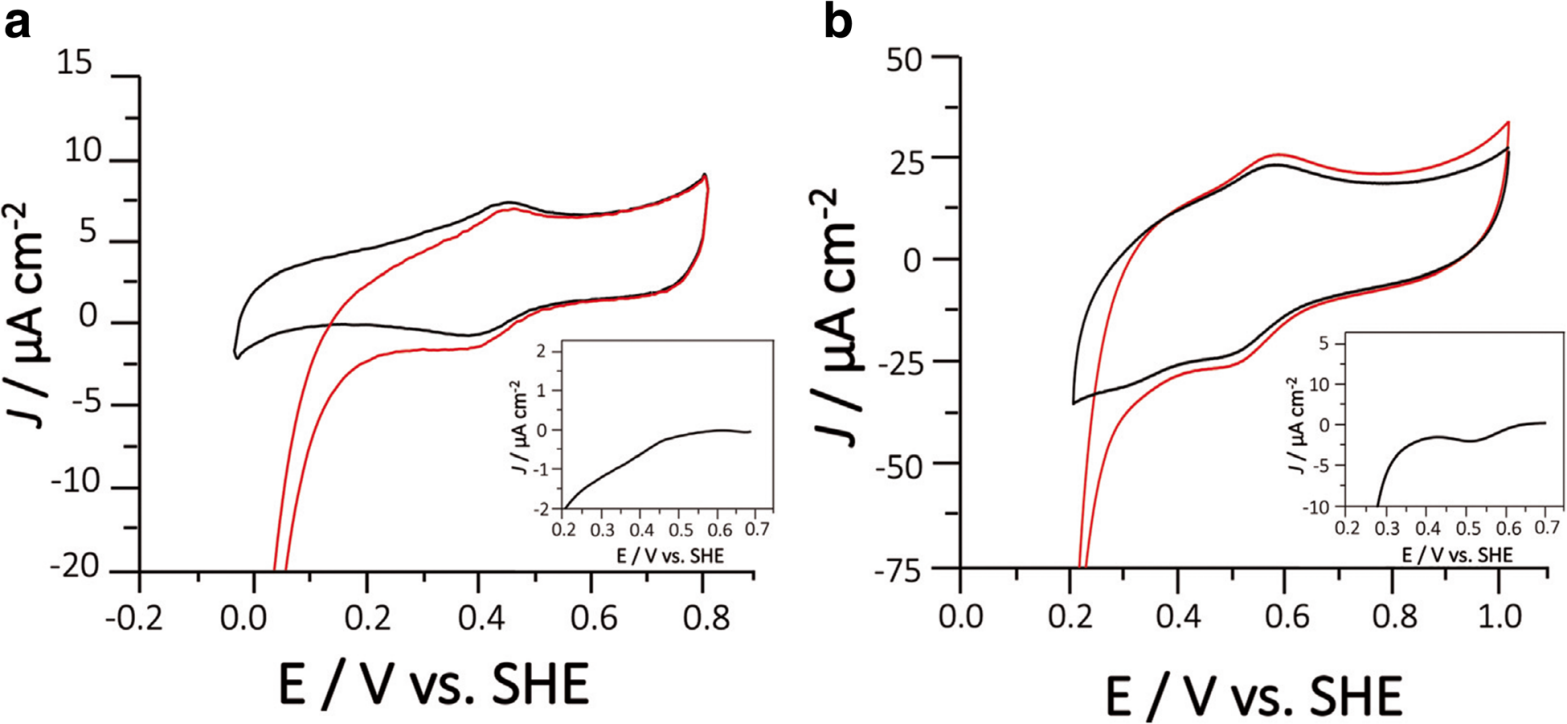
**a** Cyclic Voltammograms (CVs) recorded with *Ztr*Lac1A modified low-density GE in N_2_ (black line) and in O_2_ (red line); (inset Fig. 3a) subtracted curve in order to determine the onset of O_2_ reduction. **b** CVs recorded with GE/Os-polymer/*Zt*rLac1A under N_2_ (black line) and in O_2_ (red line) conditions. (Inset Fig. **b**) subtracted curve in order to determine the onset of O_2_ reduction. Conditions: 0.1 M acetate buffer at pH 4.0 in 0.1 M NaClO_4_; scan rate 10 mVs^− 1^

In order to study the electrocatalytic behavior of *Ztr*Lac1A, modified graphite electrode (GE) cyclic voltammograms (CVs) were collected in absence (black curve) and presence (red curve) of O_2_ in 0.1 M acetate buffer at pH 4.0, 0.1 M NaClO_4_ (Fig. 3a). It was not possible to define the onset potential for the electroreduction of O_2_ from these CVs, while this was clearly displayed in the subtracted curve at 0.569 V vs. SHE (Fig. 3). A second electroreduction process was observed at approximately 0.2 V and attributed to direct non-enzymatic oxygen reduction at the GE surface. Unmodified GE studied at the same conditions showed only electro-reduction at 0.2 V as shown in (Additional file 1: Figure S4). The peaks observed with *E*°′ 0.408 V vs. SHE (Fig. 3a) are attributed to quinone moieties at the GE as previously reported [33, 34]. The *Ztr*Lac1A modified GE electrodes do not show O_2_ electroreduction at this potential implying that there is no electrical communication between these quinone moieties and the enzyme.

Furthermore, to confirm the results, *Ztr*Lac1A was co-immobilized with an Os-redox polymer and polyethylene glycol diglycidyl ether (PEGDGE) (GE/Os/*Ztr*Lac1A) using previously reported methods [35, 36]. An Os- polymer with (*E*°′ = 0.549 V vs. SHE) was used as a surface immobilized redox mediator [37] to facilitates electron transfer between electrode surface and the T1 site of the enzyme. This mediated electron transfer is thermodynamically favorable if the *E*°‘of the polymer is more negative than *E*°’ of the T_1_ site. A polymer with *E*°′ close to that of the T1 site is desirable. This together with the highly flexible backbone and highly cationic nature of the polymer facilitate enzyme immobilization and electron transfer between the Os^2+/3+^ containing complexes, the enzyme and electrode. The polypyrrole side chains present reactive –NH_2_ groups, which are used in combination with PEGDGE to stabilize the immobilization through covalently links between the enzyme and polymer. This results in a 3D-hydrogel incorporating very high amounts of both immobilized enzyme molecules as well as mediating functionalities onto the electrode surface with easy access for substrates and products to diffuse to/from the enzyme active site. The electrochemical characterization of *Ztr*Lac1A entailed the determination of the onset potential for the oxygen electro-reduction (*E*_O2_), which is close to the formal potential of the T_1_ site (*E*_T1_) [38]. The onset potential was estimated by physically adsorbing the enzyme onto graphite electrodes (GE) with or without an Os-polymer of *E*°^′^ 0.549 V vs. Standard hydrogen electrode (SHE) previously characterized [37].

The Os-polymer facilitates transfer of electrons between the electrode and enzyme in a process known as mediated electron transfer (MET). CVs corresponding to GE/Os/*Ztr*Lac1A are shown in Fig. 3b with the subtracted curves, where the peaks observed at 0.532 V vs. SHE are attributed to the Os-polymer. Two electrocatalytic processes were observed in the presence of O_2_ with the first having its onset potential at 0.660 V vs. SHE, which is attributed to the MET between the Os-polymer and *Ztr*Lac1A. The second process starting from 0.4 V vs. SHE is attributed to the direct electroreduction of O_2_ in the surface of the GE as previously discussed.

### Phylogenetic and sequence analysis

The aligned sequences were from the ascomycetes laccases retrieved from the CAZy database and the basidiomycetes sequences from a BLASTp search of the non-redundant protein sequences database using the catalytic module of *Ztr*Lac1A (556 residues) as a query. This analysis revealed that the closest orthologues to *Ztr*Lac1A are laccases from the plant pathogens *Zymoseptoria brevis* (*Zbr*Lac1A) and *Sphaerulina musiva* (*Smu*Lac1A) sharing 98 and 69% sequence identity, respectively. In total, 221 sequences (490–650 amino acid residues, 55–75 kDa), including ascomycete and basidiomycetes laccases, were included in the alignment and phylogenetic tree.

**Fig. 4:**
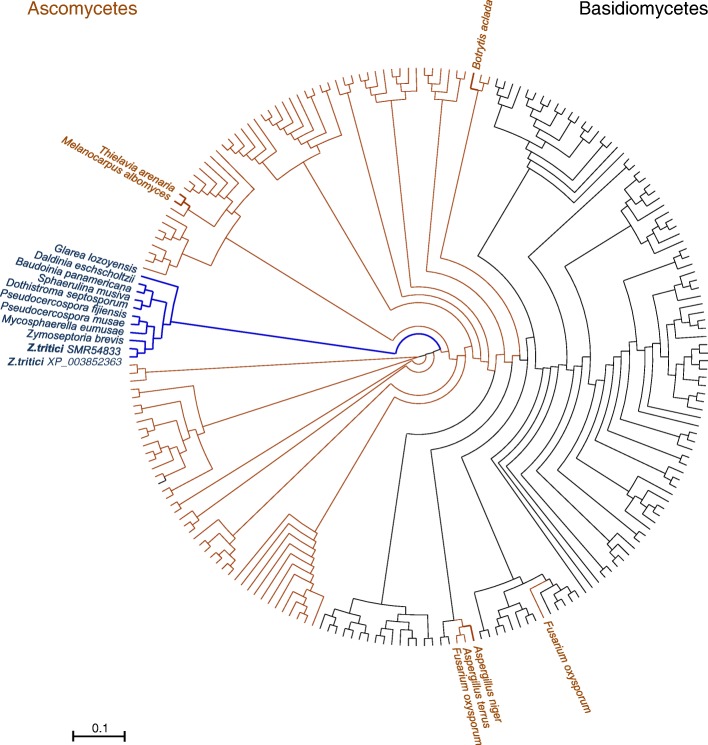
Phylogenetic analysis of *Ztr*Lac1A and related fungal laccases. The phylogram shows 221 orthologues to the catalytic module of *Ztr*Lac1A, all assigned into AA1 according to the CAZy classification. The *Septoria* cluster is highlighted in blue and the two sequences from the *Zymoseptoria tritici* species are in bold, whereas basidiomycetes sequences are in black. The only four structurally characterized ascomycete laccases are in brown. The tree illustrates the segregation of *Septoria* in a distinct clade of the phylogenetic tree consistent with the unique biochemical profile and the prevalent occurrence with a CBM20 module as opposed to other enzymes

*Ztr*Lac1A together with eight closest homologues segregate into a single branch, which is hereafter designated as the *Septoria* cluster in the phylogenetic analysis. Remarkably, six of these putative laccases share the same modular organization as *Ztr*Lac1A (Additional file 1: Figure S3a) with variations in the length of linker (12–17 amino acid residues) separating the binding and catalytic modules (Additional file 1: Table S1). The *Septoria* cluster (bold blue in Fig. 4) is adjacent to the two closest structurally characterized ascomycete laccases, *Melanocarpus albomyces* (*Mal*Lac1A) and *Thielavia arenaria* (*Tar*Lac1A), displaying 44% sequence identity to *Ztr*Lac1A (in brown). Two other structurally characterized ascomycete laccases from *Botyris aclada* (*Bac*Lac1A) and *Aspergillus niger* (*Ani*Lac1A) [39] that share 41 and 36% sequence identity to *Ztr*Lac1A, respectively, segregate in two distant clades. The basidiomycetes orthologues populate a distinct clade, which includes some intermediate ascomycete sequences from *A. niger* and *Fusarium oxysporum* (Fig. 4).

#### Distinctive structural features in ZtrLac1A compared to other fungal laccases

To analyse the structural elements responsible for segregation of the *Ztr*Lac1A in a distinct cluster, we compared these enzymes to related sequences of AA1_3 in CAZy. To assess the functional relevance of these differences, we generated homology models of *Ztr*Lac1A based on the closest structurally characterized ascomycete laccase from *M. albomyces* (*Mal*Lac1A) as a template (44% identity to *Ztr*Lac1A). A good quality model was obtained as judged by LGscore and MaxSub qualitative values of 6.18 and 0.24 calculated via the ProQ online server [40], respectively. Overall, the model was similar to *Mal*Lac1A as reflected by the root mean square deviation (RMSD) of 0.78 Å for the superimposition of 528 C_α_ backbone atoms out of 556 between these enzymes, which also shared three disulphide bridges that stabilize their structures. In *Ztr*Lac1A, the disulfide bridges between Cys303-Cys340, Cys118-Cys544, and Cys10-Cys19 at the N-terminus stabilize the fold analogous to counterparts in the *Tar*Lac1A and *Ani*Lac1A structures.

The sequence alignment between *Ztr*Lac1A and the characterized laccases revealed differences in three functionally relevant regions including loops surrounding the active site, C-terminus and copper 1 (T1) active center.

**Fig. 5:**
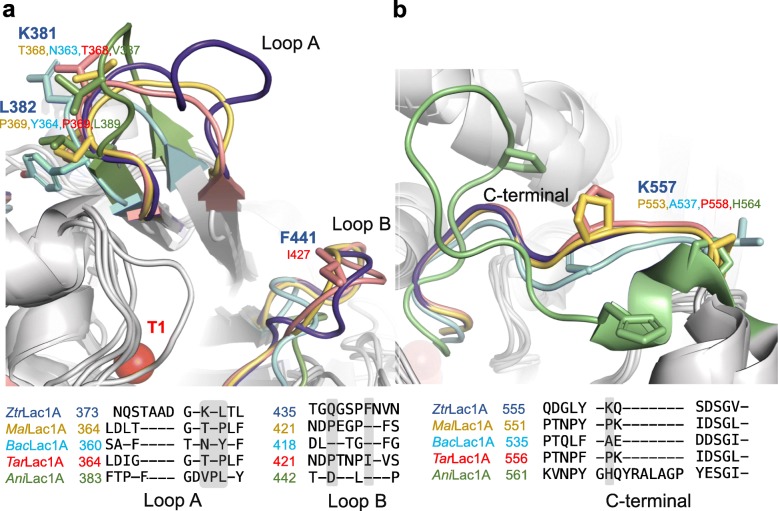
Superimposition of modeled *Ztr*Lac1A with the structures from the ascomycetes *M. albomyces* (*Mal*Lac1A, 1GW0), *T. arenaria* (*Tar*Lac1A, 3PPS), *Botyris aclada* (*Bac*Lac1A, 3SQR) and *A. niger* (*Ani*Lac1A, 5LM8). **a** The loop A flanking the T1 substrate binding site in *Ztr*Lac1A (dark blue) is variable with respect to size and sequence from the structurally characterized *Mal*Lac1A (light brown), *Tar*Lac1A (red), *Bac*Lac1A (Cyan) as well as *Ani*Lac1A (green). **b** Superposition of C-termini of *Ztr*Lac1A, *Mal*Lac1A, *Tar*Lac1A, *Bac*Lac1A and *Ani*Lac1A. The Cu 1 and Cu 2–3 are shown as red spheres. Structures were rendered using PYMOL v1.8 software (Schrödinger, LLC, Palo Alto, CA). The sequence alignment of these functionally relevant structural elements is shown and divergent segments and amino acid substitutions that involve large changes in chemistry or length of sidechains are highlighted in grey shades

The substrate-binding site in fungal laccases is defined by four loops that flank the T1 site [39]. Ascomycete laccases have elongated loops in this region compared to basidiomycetes laccases. Notably, Loop A (N373 − L384) in *Ztr*Lac1A is further elongated with two, four and six residues compared to the corresponding loops in the known structural homologues *Ani*Lac1A, *Mal*Lac1A, *Tar*Lac1A, and *Bac*Lac1A, respectively (Fig. 5a). Significant sequence substitutions are also observed in this loop, e.g. the T368, N363 and V387 in *Mal*Lac1A (and *Tar*Lac1A), *Bac*Lac1A and *Ani*Lac1A laccases are substituted with the K381 in *Ztr*Lac1A (Fig. 5a). Similarly, Loop B comprising T435 − N444 in *Ztr*Lac1A, is also elongated with up to six residues compared to *Tar*Lac1A, *Mal*Lac1A, *Bac*Lac1A as well as *Ani*Lac1A possessing the shortest B loop. Notably, a proline (P423) in *Mal*Lac1A and *Tar*Lac1A, is substituted with glutamine (Q437) in *Ztr*Lac1A (Additional file 1: Figure S5).

*Ztr*Lac1A also displays important difference at the C-terminus, with three proline residues in the structurally characterized laccases, being substituted to leucine, glutamine or lysine (Fig. 5b). Mutation of the leucine at this position to methionine was shown to reduce the activity of *Bal*Lac1A highlighting the functional importance of C-terminus [41].

## Discussion

### The first starch binding modular laccase from fungal pathogens that use apoplastic infection strategy

Carbohydrate active enzymes (CAZymes) are frequently modular featuring one or more catalytic modules (CMs) appended to non-catalytic auxiliary modules. The most common non-catalytic modules are ancillary CBMs, which potentiate the deconstruction of insoluble polysaccharides by mediating substrate targeting and prolonged contact of cognate enzymes [42]. Presence of CBMs is also observed in oxidoreductases targeting complex polysaccharides [43]. In this study, we report the properties of the first modular laccase possessing a starch-binding CBM20. This enzyme stems from the major wheat pathogen *Z. tritici* causing massive crops yield losses globally [17, 18]. *Z. tritici* has a “stealth” pathogenesis, with an apoplastic and symptomless initial growth within the plant cell wall. During later stages, loss of plant cell integrity, leakage of nutrients to the intercellular space concomitant with acceleration of fungal growth and finally necrotic lesions on leaves are typically observed [19]. Despite the significant reduction in CAZymes and CBMs [44], the genome of *Z. tritici* encodes at least 24 putative starch degrading enzymes including α-glucosidases (8 of GH31 and 1 of GH133), α-amylases (14 sequences from GH13) and a glucoamylase (GH15) (http://www.cazy.org) [45]. This genomic expansion with putative starch targeting enzymes is also reflected by the presence of seven encoded proteins possessing putative starch binding domains including four assigned into CBM20. Notably, large changes in chloroplasts are observed during infection, especially during the later stages, which revealed an expansion of chloroplasts before cell collapse [46]. The chloroplasts are the sites of starch synthesis in the leaves [46], and interestingly a recent transcriptional analysis revealed a substantial upregulation of α-amylases and oxidoreductases in the taxonomically related pine pathogen *Dothistroma septosporum* also during the later stage of infection [47]. This fungus possesses a homologue of the *Z. tritici* laccase characterized in the present study (Additional file 1: Figure S3, Additional file 1: Table S1). In fact, all the seven modular laccases possessing a CBM20 stem from taxonomically related pathogens from the order Capnodiales. Taken altogether, the evolution of starch binding laccases in this group of pathogens and the histological and transcriptional response seems to highlight an important role of starch targeting enzymes in the necrotic stage of infection. Our data provide compelling evidence for the functionality of the CBM20, which possesses moderate affinity (*K*_d_ 33 μM) typical of counterparts from amylolytic enzymes. Therefore, *Ztr*Lac1A is likely to be efficiently targeted to starch granules, but the role of the laccase and the substrates it oxidizes at the starch granule surface in vivo remain intriguing and merits further studies.

### ZtrLac1A displays a unique activity profile and sequence signatures compared to ascomycete characterized laccases

The kinetic data of *Ztr*Lac1A on the negatively charged redox mediator ABTS verified its laccases functionality (Table 1). Interestingly, the *K*_m_ of this enzyme on ABTS was 60% lower than that measured for the closest related ascomycete laccases *Mal*Lac1A (Table 1). While, *Mal*Lac1A displays an increasing affinity and efficiency on the more apolar phenolic mediators DMP and syringaldazine compared to ABTS [29], *ZtrLac1A* was almost inactive on these substrates (Table 1). This sequence signature is also consistent with changes in the sequence and length of loops flanking the T1 binding site, where the substrate binding and oxidation occurs as compared to *Mal*Lac1A and other currently characterized ascomycete laccases (Fig. 5, Additional file 1: Figure S5). Notably, the loops flanking the active site of *Ztr*Lac1A, exhibit a lower content of negatively charged and apolar residues compared to structurally characterized laccases. These changes, together with the elongation of loop A with four amino acid residues including a lysine (Fig. 5a), are consistent with a more positive electrostatics and occlusion of the active site in *Ztr*Lac1A. These substitutions may provide a rationale for better affinity of the negatively charged ABTS and the lack of activity on the two other less polar mediators. The C-terminal loop of laccases acts as a plug that occludes the T2 and T3 sites, where the second substrate O_2_ binds [29]. A single mutation of the C-terminal residue (L559A) and the deletion of the four C-terminal residues of *Mal*Lac1A caused a severe activity drop of the enzyme [29]. We have also observed important substitutions in this C-terminal segment making it more flexible compared to characterized counterpart due to the loss of three closely located proline residues (Fig. 5b).

Another interesting difference is a conserved residue close to the Cu ligands at the T1 site (Additional file 1: Figure S6). This residue is either a phenylalanine as in *Ztr*Lac1A, or a leucine as in characterized ascomycete laccases (Additional file 1: Figure S6), but methionines are also observed in a few non-characterized ascomycete laccases. Site-directed mutagenesis of Cu ligands have shown large effects on the redox potential [39, 48]. According to the current classification, laccases are arranged in three groups: low (340–490 mV), middle (470–710 mV) and high potential (730–780 mV) [49]. The difference in the T_1_ potentials depends strongly on the axial Cu-ligands of the T1 site being methionine for low potential, leucine for middle and phenylalanine for high potentials [49].

The bioelectrochemical O_2_ reduction by *Ztr*Lac1A immobilized on GE electrodes gives an estimate of the potential of T1, being 0.569 V vs. SHE when the electrons are transferred directly from the electrode to the T1 site of the enzyme. The successfully mediated electroreduction observed for GE/Os/*Ztr*Lac1A is an indication of the closeness of the *E*^0^’ of T_1_ to the one of the Os-polymer used (0.55 V). The assignment of the onset to be from the T_1_ site is based on previously published results reporting the electro-reduction of O_2_ at carbon modified electrodes with “blue” MCO [50–52]. Subsequently, electron transfer proceeds internally through the T_2_/T_3_ multicopper site, where the electroreduction of O_2_ takes place.

*Ztr*Lac1A is thus classified as a middle redox potential accordingly, which is in agreement with previous values reported for the few other ascomycete characterized laccases [48].

## Conclusions

*Ztr*Lac1A displays an atypical substrate preference, but shows a typical redox potential range compared to ascomycetes characterized counterparts. This first demonstration of activity of a modular laccase with a starch binding module offers an attractive possibility for reversible immobilization of laccases on cheap material like starch or cellulose, and allows targeting of these enzymes without using classical covalent immobilization techniques that typically reduce enzymatic activity. Finally, further studies are needed to get insight into the role of this enzyme in the pathogenic life cycle of fungi from the order Capnodiales and to identify physiologically relevant substrates.

## Additional file

Additional file 1: **Table S1.** Septoria cluster laccases. **Figure S1.** Chemical structure of the osmium polymer. **Figure S2.** Activity screening on aromatic (mainly phenolics) susbtrates. **Figure S3.** ZtrLac1A primary structure and purification. **Figure S4.** Cyclic voltammograms. **Figure S5.** Sequence alignment of ZtrLac1A and homologues from AA1_3. **Figure S6.** Architecture of Cu1 copper site in characterized ascomycetes laccases. (PDF 747 kb)

## Data Availability

All data generated or analyzed during this study are included in this published article and its supplementary information files.

## Abbreviations

2,6-DMP: 2,6-dimethoxyphenol
ABTS: 2,2′-azino-bis(3-ethylbenzothiazoline-6-sulfonic acid)
*Ani*Lac1A: *Aspergillus niger* laccase
*Bac*Lac1A: *Botyris aclada* laccase
CBM: Carbohydrate binding module
CMs: Catalytic modules
CV: Cyclic voltammograms
GE: Graphite electrode
*Mal*Lac1A: *Melanocarpus albomyces* laccase
MCOs: Multicopper oxidases
SHE: Standard hydrogen electrode
STB: *Septoria tritici* blotch
*Tar*Lac1A: *Thielavia arenaria* laccase
*Ztr*Lac1A: *Zymoseptoria tritici* laccase
β-CD: β-cyclodextrin

## References

[1] Solomon EL, Sundaram UM, Machonkin TE (1996). Multicopper oxidases and oxygenases. Chem Rev.

[2] Giardina P, Faraco V, Pezzella C, Piscitelli A, Vanhulle S, Sannia G (2010). Laccases: a never-ending story. Cell Mol Life Sci.

[3] Solomon EI, Heppner DE, Johnston EM, Ginsbach JW, Cirera J, Qayyum M, Kieber-Emmons MT, Kjaergaard CH, Hadt RG, Tian L (2014). Copper active sites in biology. Chem Rev.

[4] Solomon EI, Chen P, Metz M, Lee SK, Palmer AE (2001). Oxygen binding, activation, and reduction to water by copper enzymes. Angew Chem Int Ed Engl.

[5] Kamitaka Y, Tsujimura S, Kataoka K, Sakurai T, Ikeda T, Kano K (2007). Effects of axial ligand mutation of the type I copper site in bilirubin oxidase on direct electron transfer-type bioelectrocatalytic reduction of dioxygen. J Electroanalytical Chem.

[6] Reinhammar BRM (1972). Oxidation-reduction potentials of the electron acceptors in laccases and stellacyanin. Biochim Biophys Acta.

[7] Rodjers CJ, Blandford CF, Giddens SR, Skamnioti P, Armstrong FA, Gurr SJ (2010). Designer laccases: a vogue for high-potential fungal enzymes?. Trends Biothechnol.

[8] Claus H (2004). Laccases: structure, reactions, distribution. Micron..

[9] Levasseur A, Drula E, Lombard V, Coutinho PM, Henrissat B (2013). Expansion of the enzymatic repertoire of the CAZy database to integrate auxiliary redox enzymes. Biotechnol Biofuels.

[10] Kiiskinen LL, Viikari L, Kruus L (2002). Purification and characterisation of a novel laccase from the ascomycete *Melanocarpus albomyces*. Appl Microbiol Biotechnol.

[11] Baldrian P (2006). Fungal laccases - occurrence and properties. FEMS Microbiol.

[12] Slomczynski D, Nakas JP, Tanenbaum SW (1995). Production and characterization of laccase from *Botrytis cinerea*. Appl Environ Microbial.

[13] Iyer G, Chattoo BB (2003). Purification and characterization of laccase from the rice blast fungus, *Magnaporthe grisea*. FEMS Microbiol Lett.

[14] Canero Cordoba D, Roncero MIG (2007). Functional analyses of laccase genes from *Fusarium oxysporum*. Amercian phytopathol. Soci..

[15] O’Driscoll A, Kildea S, Doohan F, Spink J, Mullins E (2014). The wheat-Septoria conflict: a new front opening up?. Trends Plant Sci.

[16] Torriani SFF, Melichar JPE, Mills C, Pain N, Sierotzki H, Courbot M (2015). *Zymoseptoria tritici:* a major threat to wheat production, integrated approaches to control. Fungal Genet Biol.

[17] Fones H, Gurr S (2015). The impact of *Septoria tritici* blotch disease on wheat: an EU perspective. Fungal Genetic Biol.

[18] Jørgensen Lise Nistrup, Hovmøller Mogens Støvring, Hansen Jens Grønbæk, Lassen Poul, Clark Bill, Bayles Rosemary, Rodemann Bernd, Flath Kerstin, Jahn Margot, Goral Tomas, Jerzy Czembor J, Cheyron Philip, Maumene Claude, De Pope Claude, Ban Rita, Nielsen Ghita Cordsen, Berg Gunilla (2014). IPM Strategies and Their Dilemmas Including an Introduction to www.eurowheat.org. Journal of Integrative Agriculture.

[19] Dean R, Van Kan JA, Pretorious ZA, Hammond-Kosack KE, Di Pietro A, Spanu PD, Rud JJ, Dickman M, Kahmann R, Elis J, Foster GD (2012). The top 10 fungal pathogens in molecular plant pathology. Mol Plant Pathol.

[20] Windels CE (2000). Economic and social impacts of *Fusarium* head blight: changing farms and rural communities in the northern Great Plains. Phytopathology..

[21] Howard RJ, Valent B (1996). Breaking and entering: host penetration by the fungal rice blast pathogen *Magnaporthe grisea*. Annu Rev Microbiol.

[22] Goodwin Stephen B., Ben M'Barek Sarrah, Dhillon Braham, Wittenberg Alexander H. J., Crane Charles F., Hane James K., Foster Andrew J., Van der Lee Theo A. J., Grimwood Jane, Aerts Andrea, Antoniw John, Bailey Andy, Bluhm Burt, Bowler Judith, Bristow Jim, van der Burgt Ate, Canto-Canché Blondy, Churchill Alice C. L., Conde-Ferràez Laura, Cools Hans J., Coutinho Pedro M., Csukai Michael, Dehal Paramvir, De Wit Pierre, Donzelli Bruno, van de Geest Henri C., van Ham Roeland C. H. J., Hammond-Kosack Kim E., Henrissat Bernard, Kilian Andrzej, Kobayashi Adilson K., Koopmann Edda, Kourmpetis Yiannis, Kuzniar Arnold, Lindquist Erika, Lombard Vincent, Maliepaard Chris, Martins Natalia, Mehrabi Rahim, Nap Jan P. H., Ponomarenko Alisa, Rudd Jason J., Salamov Asaf, Schmutz Jeremy, Schouten Henk J., Shapiro Harris, Stergiopoulos Ioannis, Torriani Stefano F. F., Tu Hank, de Vries Ronald P., Waalwijk Cees, Ware Sarah B., Wiebenga Ad, Zwiers Lute-Harm, Oliver Richard P., Grigoriev Igor V., Kema Gert H. J. (2011). Finished Genome of the Fungal Wheat Pathogen Mycosphaerella graminicola Reveals Dispensome Structure, Chromosome Plasticity, and Stealth Pathogenesis. PLoS Genetics.

[23] Mano N, Kim HH, ZhangY, Heller A (2002). An oxygen cathode operating in a physiological solution. J Am Chem Soc.

[24] Nekiunaite L, Isaksen T, Vaaje-Kolstad G, Abou Hachem M (2016). Fungal lytic polysaccharide monooxygenases bind starch and β-cyclodextrin similarly to amylolytic hydrolases. FEBS Lett.

[25] Niku-Paavola ML, Karhunen E, Salola P, Raunio V (1988). Ligninolytic enzymes of the white-rot fungus *Phlebia radiate*. Biochem J.

[26] Shleev S, Jarosz-Wilkolazka A, Khalunina A, Morozova O, Yaropolov A, Ruzgas T, Gorton L (2005). Direct heterogeneous electron transfer reactions of laccases from different origins on carbon electrodes. Bioelectrochemistry..

[27] Altschul SF, Madden TL, Schäffer AA, Zhang J, Zhang Z, Miller W, Lipman DJ (1997). Gapped BLAST and PSI-BLAST: a new generation of protein database search programs. Nucleic Acids.

[28] Huson DH, Scornavacca C (2012). Dendroscope 3: an interactive tool for rooted phylogenetic trees and networks. Syst Biol.

[29] Andberg M, Hakulinen N, Auer S, Saloheimo M, Koivula A, Rouvinen J, Kruus K (2009). Essential role of the C-terminus in *Melanocarpus albomyces* laccase for enzyme production catalytic properties and structure. FEBS J.

[30] Borodina I, Jensen BM, Wagner T, Abou Hachem M, Sondergaard I, Poulsen LK (2011). Expression of enzymatically inactive wasp venom phospholipase A1 in *Pichia pastoris*. PLoS One.

[31] Jeoh T, Michener W, Himmel ME, Decker SR, Adney WS (2008). Implications of cellobiohydrolase glycosylation for use in biomass conversion. Biotechnol Biofuels.

[32] Christiansen C, Abou Hachem M, Janecek S, Viksø-Nielsen A, Blennow A, Svensson B (2009). The carbohydrate-binding module family 20-diversity, structure, and function. FEBS J.

[33] Swain Greg M. (2007). Solid Electrode Materials. Handbook of Electrochemistry.

[34] McCreery RL (2008). Advanced carbon electrode materials for molecular electrochemistry. Chem Rev.

[35] Heller A (1990). Electrical wiring of redox enzymes. Acc Chem.

[36] Zafar MN, Tasca F, Boland S, Kujawa M, Patel I, Peterbauer CK, Leech D, Gorton L. Wiring of pyranose dehydrogenase with osmium polymers of different redox potentials. Bioelectrochemistry. 2010;8038–42. 10.1016/j.bioelechem.2010.04.002 20466600

[37] Kavanagh P, Jenkins P, Leech D (2008). Electroreduction of O_2_ at a mediated *Melanocarpus albomyces* laccase cathode in a physiological buffer. Electrochem Commun.

[38] Dos Santos L, Climent V, Blandford CF, Armstrong FA (2010). Mechanistic studies of the ‘blue’ cu enzyme, bilirubin oxidase, as a highly efficient electrocatalyst for the oxygen reduction reaction. Phys Chem Chem Phys.

[39] Ferraroni M, Westphal AH, Borsari M, Tamayo-Ramos JA, Briganti F, de Graaff LH, van Berkel WJH (2017). Structure and function of *Aspergillus niger* laccase McoG. Biocatalysis..

[40] Wallner B, Elofsson A (2003). Protein Sci.

[41] Osipov E, Polyakov K, Kittl R, Shleev S, Dorovatovsky TT, Hann S, Ludwig R, Popov V (2014). Effect of the L499M mutation of the ascomycetous *Botrytis aclada* laccase on redox potential and catalytic properties. Acta Crystallogr D Biol Crystallogr.

[42] Gilbert HJ, Knox JP, Boraston AB (2013). Advances in understanding the molecular basis of plant cell wall polysaccharide recognition by carbohydrate-binding modules. Curr Opin Struct Biol.

[43] Nekiunaite L, Arntzen M, Svensson B, Vaaje-Kolstad G, Abou Hachem M (2016). Lytic polysaccharide monooxygenases and other oxidative enzymes are abundantly secreted by *Aspergillus nidulans* grown on different starches. Biotechnol Biofuel.

[44] do Amaral AM, Antoniw J, Rudd H-KKE (2012). Defining the predicted protein Secretome of the fungal wheat leaf pathogen *Mycosphaerella graminicola*. PLoS One.

[45] Kema GHJ, Yu DZ, Rijkenberg FHJ, Shaw MW, Baayen RP (1996). Histology of the pathogenesis of *Mycosphaerella graminicola* in wheat. Phytopathology..

[46] Zeeman SC, Delatte T, Messerli G, Umhang M, Stettler M, Mettler T, Streb S, Reinhold H, Kötting O (2007). Starch breakdown: recent discoveries suggest distinct pathways and novel mechanisms. Funct Plant Biol.

[47] Bradshaw RE, Guo YD, Sim AD, Kabir MS, Chettri P, Ozturk IK, Hunziker L, Ganley RJ, Cox MP (2016). Genome-wide gene expression dynamics of the fungal pathogen *Dothistroma septosporum* throughout its infection cycle of the gymnosperm host *Pinus radiate*. Mol Plant Pathol.

[48] Durão P, Chen Z, Silva CS, Soares CM, Pereira MM, Todorovic S, Hildebrandt P, Bento I, Lindley PF, Martins LO (2008). Proximal mutations at the type 1 copper site of CotA laccase: spectroscopic, redox, kinetic and structural characterization of I494A and L386A mutants. Biochem J.

[49] Shleev S, Tkac J, Christenson A, Ruzgas T, Yaropolov AI, Whittaker JW, Gorton L (2005). Direct electron transfer between copper-containing proteins and electrodes. Biosens Bioelectron.

[50] Berezin IV, Bogdanovskaya VA, Varfolomeev SD, Tarasevich MR, Yaropolov AI (1978). Bioelectrocatalysis. Equilibrium oxygen potential in the presence of laccase. Dokl Akad Nauk SSSR.

[51] Yaropolov AI, Kharybin AN, Emnéus J, Marko-Varga G, Gorton L (1996). Electrochemical properties of some copper-containing oxidases. Bioelectrochem Bioenerg.

[52] Christenson A, Dimcheva N, Ferapontova EE, Gorton L, Ruzgas T, Stoica L, Shleev S, Yaropolov AI, Haltrich D, Thorneley RNF, Aust SD (2004). Direct electron transfer between ligninolytic redox enzymes and electrodes. Electroanalysis..

