# Supplementary material for: A novel starch-binding laccase from the wheat pathogen Zymoseptoria tritici highlights the functional diversity of ascomycete laccases
Source: BMC Biotechnol. 2019 Aug 19;19:61. doi: 10.1186/s12896-019-0552-4 (PMC6700816; doi:10.1186/s12896-019-0552-4)
Supplement: Supplementary file 1 — Table S1. Septoria cluster laccases. Figure S1. Chemical structure of the osmium polymer. Figure S2. Activity screening on aromatic (mainly phenolics) susbtrates. Figure S3. ZtrLac1A primary structure and purification. Figure S4. Cyclic voltammograms. Figure S5. Sequence alignment of ZtrLac1A and homologues from AA1_3. Figure S6. Architecture of Cu1 copper site in characterized ascomycetes laccases. (PDF 747 kb) [file 12896_2019_552_MOESM1_ESM.pdf]

A novel starch binding laccase from the wheat pathogen *Zymoseptoria tritici* highlights the functional diversity of ascomycete laccases

Majid Haddad Momeni[a], Paolo Bollella[b,c], Roberto Ortiz[d], Esben Thormann[d], Lo Gorton[b], Maher Abou Hachem[a]

[a] Department of Biotechnology and Biomedicine, Technical University of Denmark, Søltofts Plad, 2800 Kgs. Lyngby, Denmark

[b] Department of Analytical Chemistry/Biochemistry, Lund University, P.O. Box 124, 221 00 Lund, Sweden

[c] Department of Chemistry and Drug Technologies, Sapienza University of Rome, Piazzale Aldo Moro 5, 00185, Rome, Italy

[d] Department of Chemistry, Technical University of Denmark, Kemitorvet 207, 2800 Kgs. Lyngby, Denmark

Corresponding author
Maher Abou Hachem
Protein Glycoscience and Biotechnology,
Department of Biotechnology and Biomedicine, Technical University of Denmark,
Søltofts Plads, 2800 Kgs. Lyngby, Denmark
Email: maha@bio.dtu.dk

Table S1 List of *Zymoseptoria tritici* (*Ztr*Lac1A) and 6 other homologues with bimodular organizations

	Locus number	Length	Identity (Catalytic domain)	CBM	Linker length
Zymoseptoria tritici, *Ztr*Lac1A	XP003852363	723	100%	110	12
Zymoseptoria brevis, *Zbr*Lac1A	KJX94131	723	99%	110	12
Sphaerulina musiva, *Smu*Lac1A	XP_016758816	742	69%	108	32
Pseudocercospora musae, *Pmu*Lac1A	KXT09849	746	68%	107	32
Mycosphaerella eumusae, *Meu*Lac1A	KXS98121	719	69%	107	17
Pseudocercospora fijiensis, *Pfi*Lac1A	XP_007930185	587	68%	--	--
Dothistroma septosporum, *Dse*Lac1A	EME38505	729	68%	107	12
Daldinia eschscholtzii, *Des*Lac1A	AEX97872	418	60%	--	--
Baudoinia panamericana, *Bpa*Lac1A	XP_007679364	703	62%	108	17

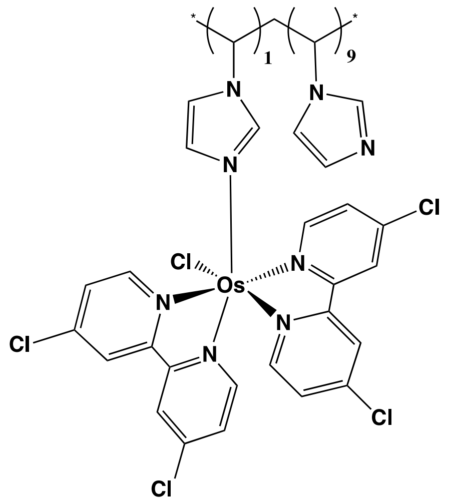

Fig. S1 Chemical structure of the osmium polymer [Os(4,4’-dichloro-2,2’-bipyridine)2(poly vinylimidazole)10Cl]·Cl with formal potentials *E*°’= 0.549 V *vs.* SHE

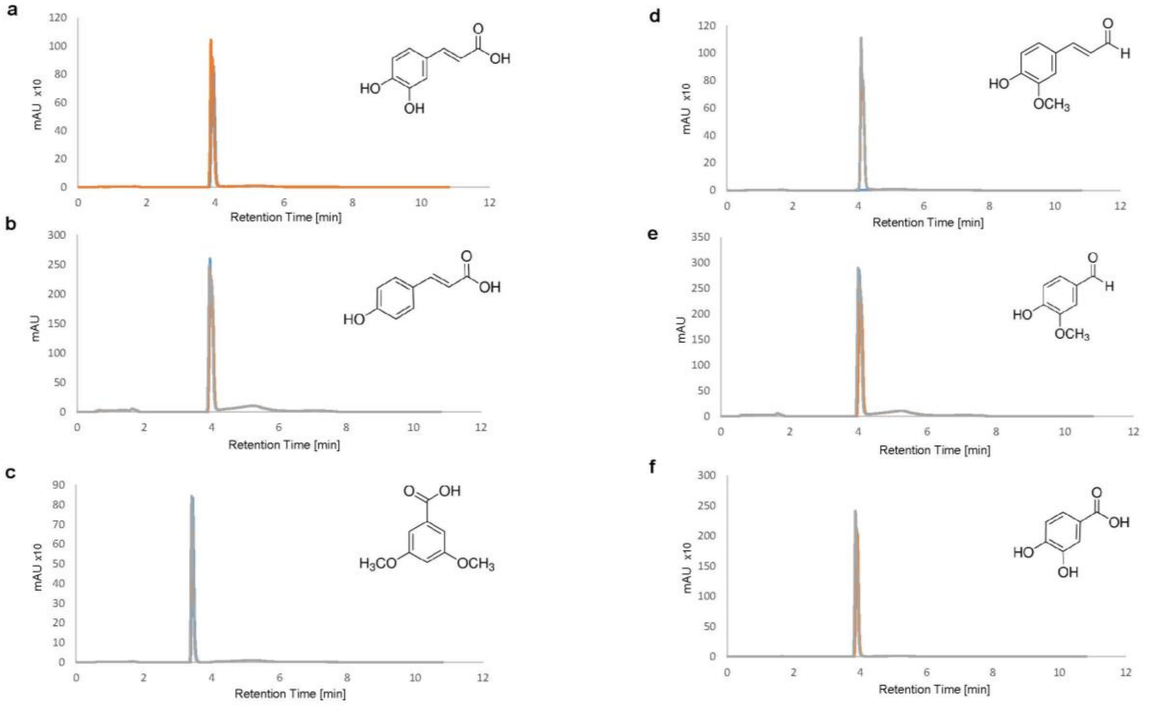

Fig. S2 HPLC ultimate profiles of different aromatic substrates assayed for *Ztr*Lac1A activity screening. The substrates are: (**a**) caffeic acid, (**b**) *p*-coumaric acid, (**c**) 3,5 dimethoxybenzoic acid, (**d**) coniferyl aldehyde, (**e**) vanillin and (**f**) 3,4 dihydroxybenzoic acid. All reactions were conducted in 0.5 ml for 45 and 90 min depicted in orange and blue respectively and controls are shown in grey. The reaction mixtures were analyzed by high performance liquid chromatography (HPLC3000), using reversed phase column C-18 (Kinetex LC column 100 mm x 4.6 mm, paricle size 2.6 µm). The elution was performed using a gradient of solvent A: water/TFA (100:0.01; v/v), solvent B: acetonitrile/TFA (100:0.01; v/v) and detection was conducted at 285 and 322 nm. The

steps of gradient were linear from 0-4% of B for 5 min, 4-30% of B for 2 min and 30-100% of B for 2 min eluted at flowrate of 1.0 ml/min at room temperature

a

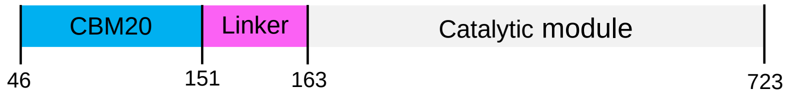

M R L H S I F N F L A L A A P I F A T V L P S Y D Y S Q R S E E V S A Q H L E S R Q S N A G
S I P V T F N L L A Q T G W G E S I Q V A G S T D E L G N W A A A N A V A L T A A Q Y T A G
N P L W S I T V D L P V G Q S F S Y K F I R I S V D G T V I W E D D P D R F Y T V P G N G
A T A A T I S N S W Q A R P **T T S** A **T** R **T** A **T S T** A P I A S S T C T N G P T S R G C W T S P
F S I N T D F D Q N W P I T G R T V S Y N F D I T **N** I T L A P D G F A R P V F A V N G Q Y P
G P T I F A N W G D T I Q V T V Q N S L Q H **N** G T S I H W H G M R M Y H A N G Q D G V P
G V T E C P I A P G R S K T Y T L L A T Q Y G T S W Y H S H F S S Q Y G D G I L G P I V I N
G P A S A N Y D I D L G A L P M T D W Y Y P T V M T T A A R A M H A N A L A P T A D N G L I
N G T M V S T S G N R G A Y A K T T L T A G K K H R L R F I N T S V D N H F M V G L D G
H I M T V I S A D F V P V E P Y **N** A S W I F I G I G Q R Y D V V I S A D Q T P G R Y W F R A
E V Q D S A G C G A N F Q N G N I R S I F S Y S S L P S N T P T L P T S S P T P Y T Q R C
S D E T S L T P V W N S Y V P R G R I P D A S S F S E L S I A I **N** Q S T A A D G K L T L Y W
Q V **N** G T P L R A D W E K P S V M T V R D G K T F P K A S N M I T F D D D D E D E W T Y
I L I T T G Q G S P F N V N I P H P I H L H G H D F Y V L G T G T S A W R D A D R Q G L N
Y E D P V R R D V A M L P S E G W L A I A W R R D N P G V W L V H C H I A W H A D E G F
A V Q F L E S P D K M L D V A P L P G D F D D Q C A A W S D F Y Q D G L Y K Q S D S G V

b

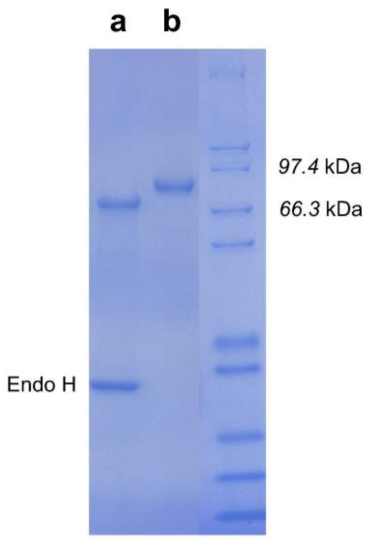

Fig. S3 (**a**) Laccase catalytic module, carbohydrate binding module and linker are colored in grey, blue and pink, respectively. Asparagine residues predicted to be *N*-glycosylated are colored in blue and those with more than 0.6 potential indicated with underline. (**b**) Purified *Ztr*Lac1A analyzed using SDS-PAGE after EndoH treatment A and before EndoH treatment B. The Mark 12 protein standard (Invitrogen) is in right lane

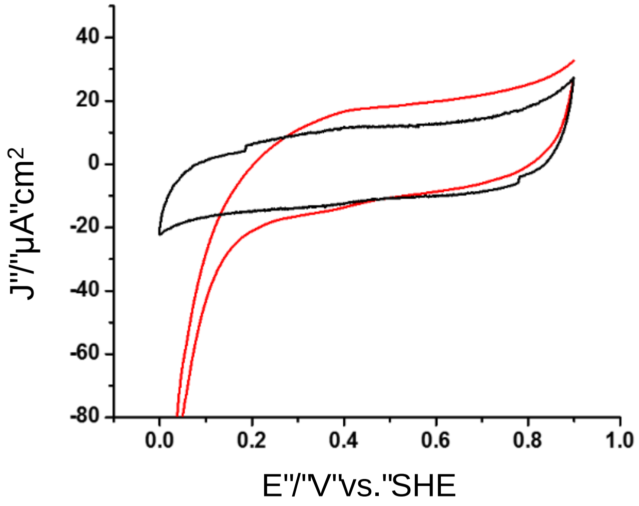

Fig. S4 Voltammograms recorded with naked graphite low-density electrode in N_2 (black line) and in the presence of O_2 (red line); Conditions: 0.1 M acetate buffer pH 4 in 0.1 M $NaClO_4$; scan rate 10 mVs^{-1}

a

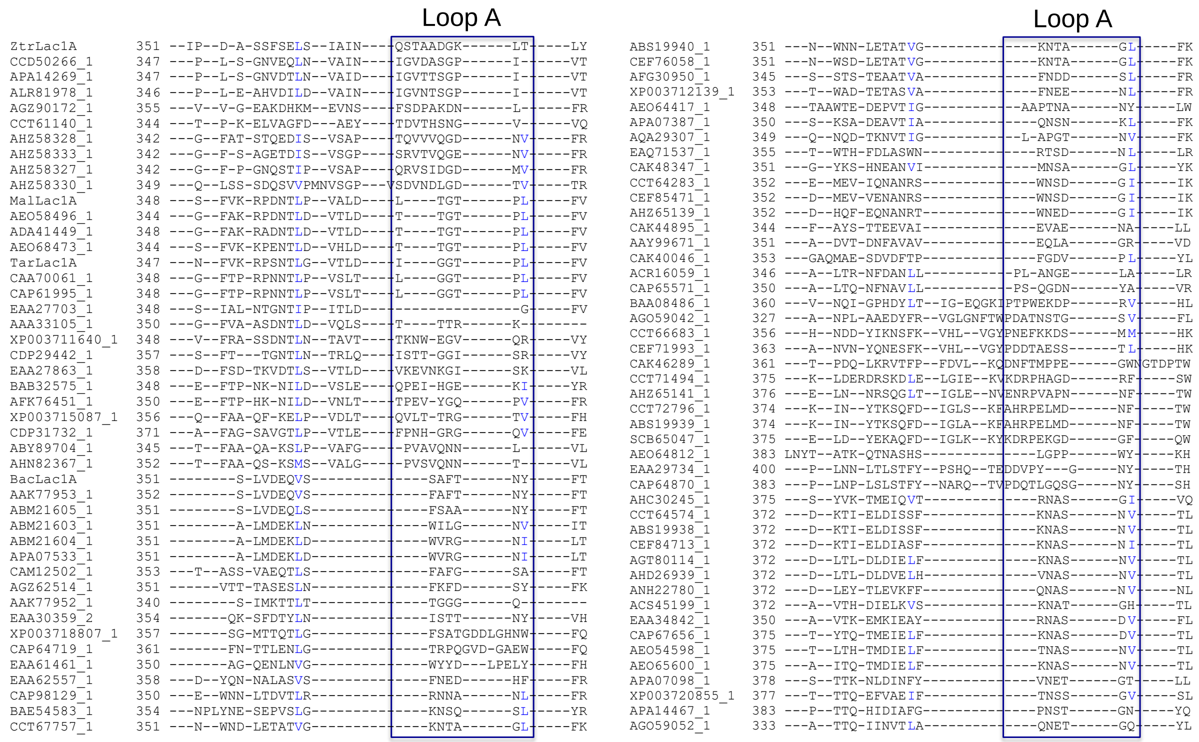

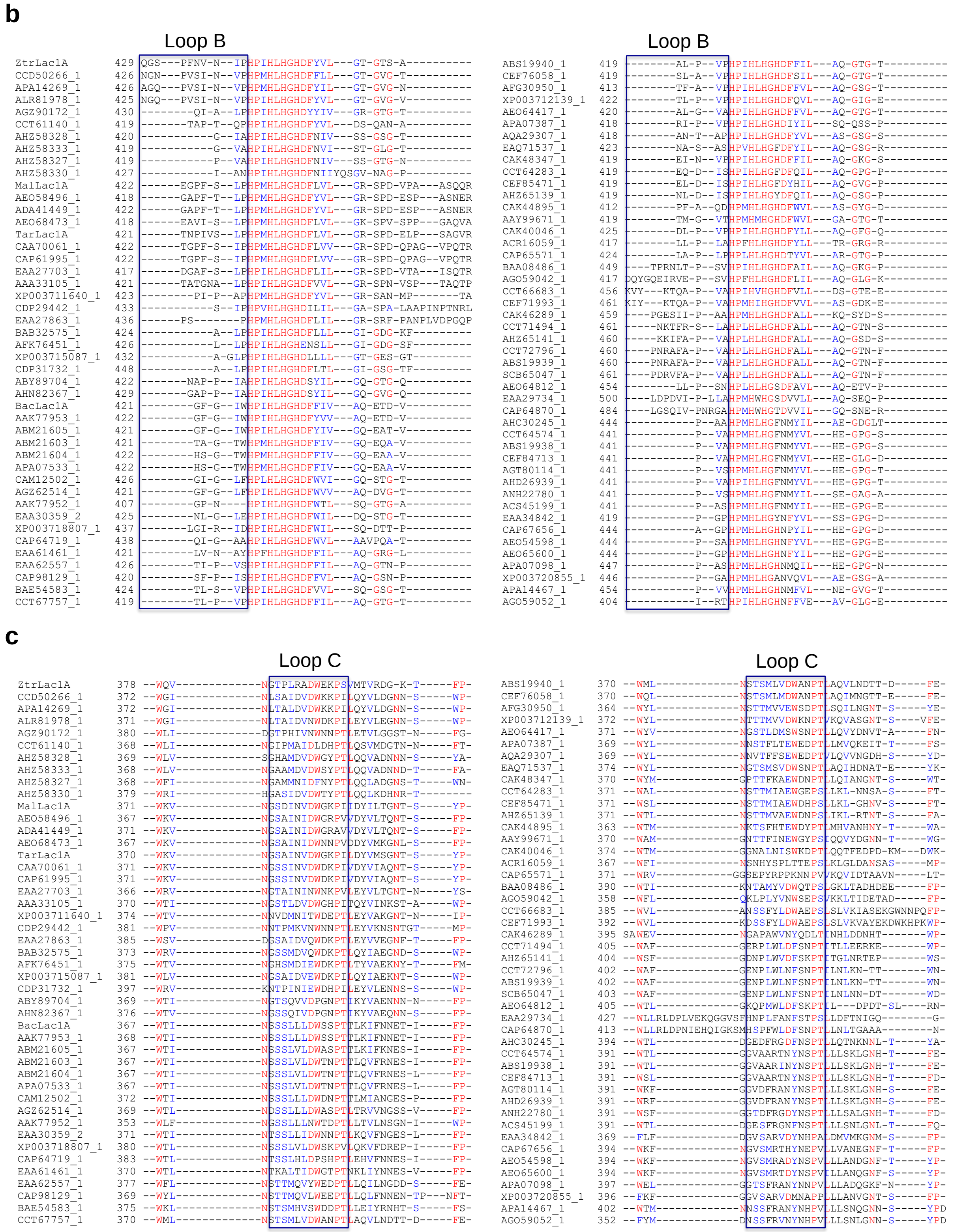
b
Loop B
Loop B
c
Loop C
Loop C

d

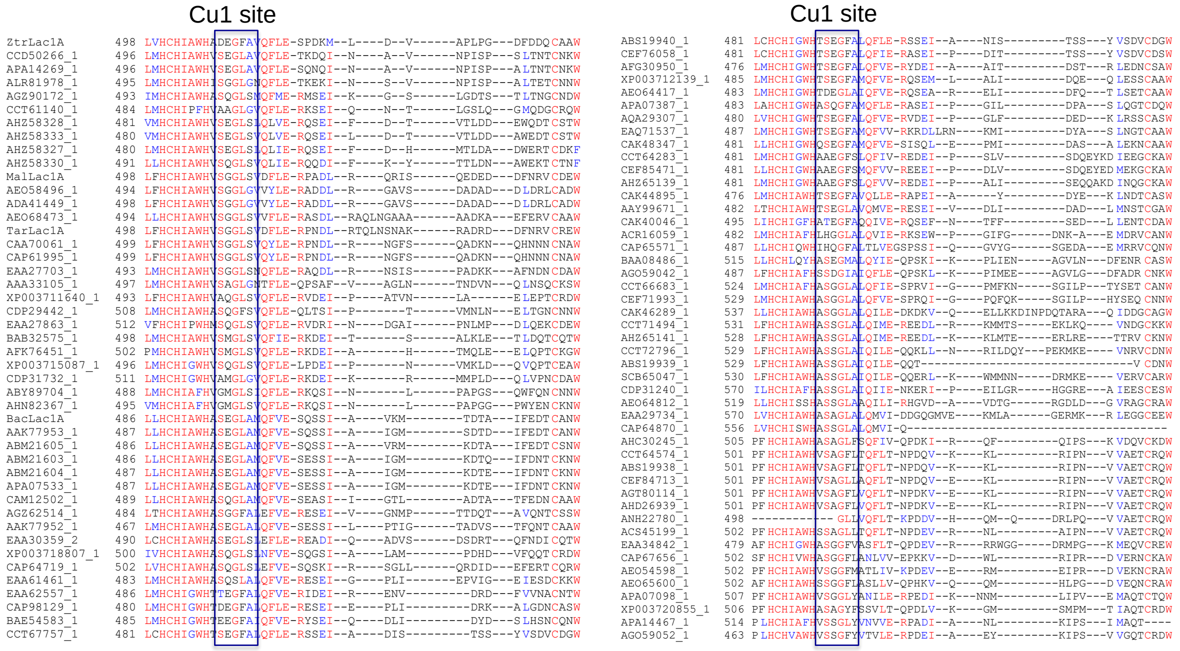

e

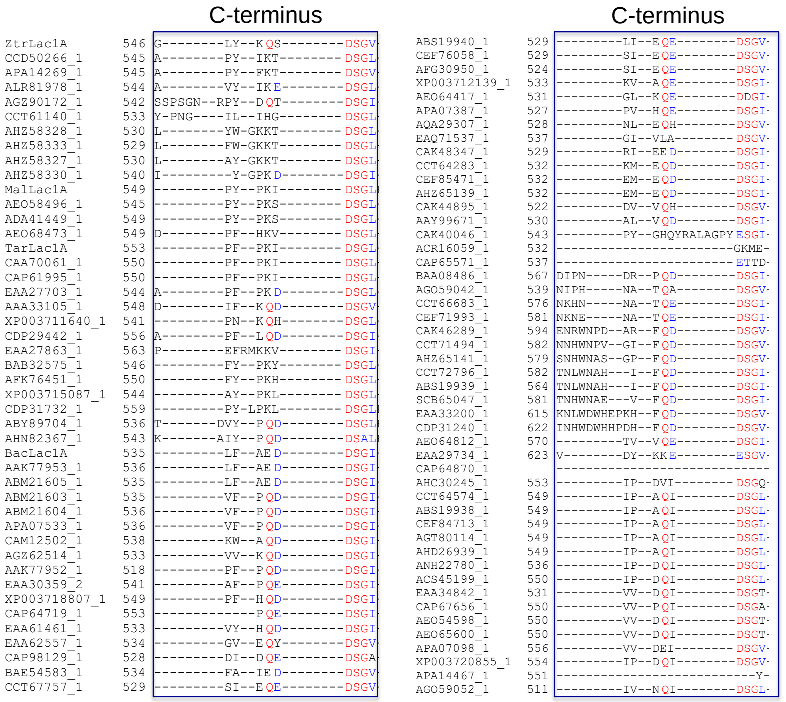

Fig. S5 Sequence alignment of *Ztr*Lac1A (catalytic module) along representative members of AA1 subfamily 3 deposited in CAZy database. All three loops A, B, C, Cu1 site ligands as well as the C-terminus regions are depicted in boxes. Fully conserved residues are in red letters, semi-conserved in blue and non-conserved in black. Accession numbers are shown except *Ztr*Lac1A, *Mal*Lac1A, *Tar*Lac1A and *Bac*Lac1A

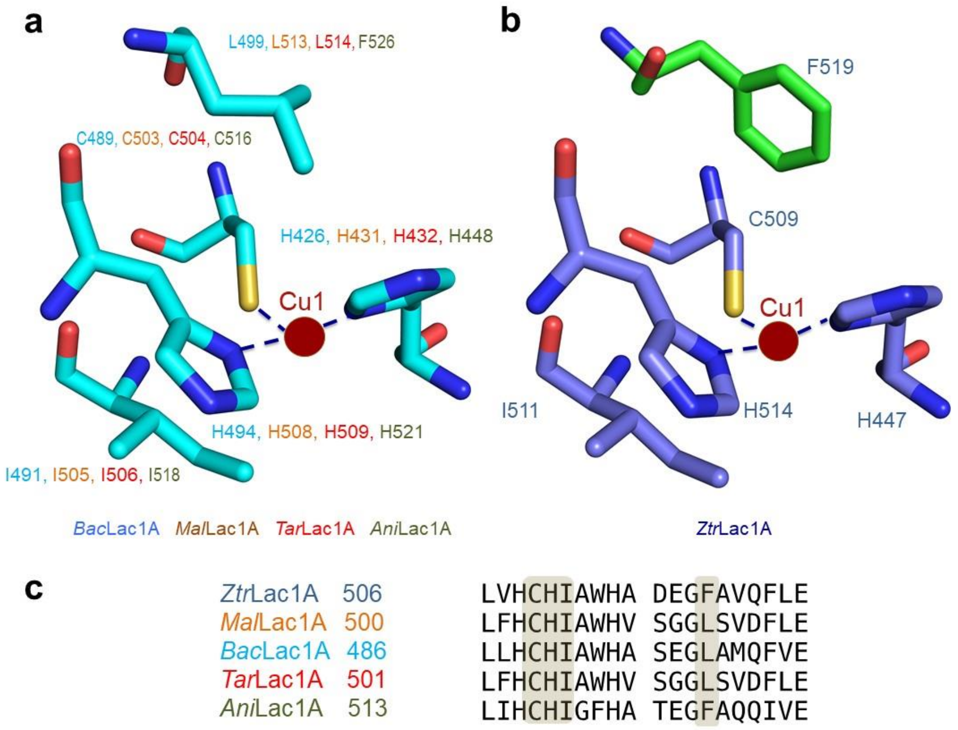

Fig. S6 Architecture of Cu1 copper site in *Bac*Lac1A, *Mal*Lac1A, *Tar*Lac1A and *Ani*Lac1A (**a**) and *Ztr*Lac1A (**b**). The Cu1 copper ions are depicted as brown spheres and their coordination bonds are shown in black dashed lines. The structure of *Bac*Lac1A PDB entry 3SQR was used [41]. (**c**) Structural sequence alignment of *Ztr*Lac1A with *Mal*Lac1A, *Tar*Lac1A, *Bac*Lac1A and *Ani*Lac1A

References

41. Osipov E, Polyakov K, Kittl R, Shleev S, Dorovatovsky P, Tikhonova T, Hann S, Ludwig R, Popov V. Effect of the L499M mutation of the ascomycetous *Botrytis aclada* laccase on redox potential and catalytic properties. Acta Crystallogr D Biol Crystallogr. 2014;70:2913−2923.
